# SARS-CoV-2 Antibody Binding and Neutralization in Dried Blood Spot Eluates and Paired Plasma

**DOI:** 10.1128/Spectrum.01298-21

**Published:** 2021-10-20

**Authors:** Hannah L. Itell, Haidyn Weight, Carolyn S. Fish, Jennifer K. Logue, Nicholas Franko, Caitlin R. Wolf, Denise J. McCulloch, Jared Galloway, Frederick A. Matsen, Helen Y. Chu, Julie Overbaugh

**Affiliations:** a Human Biology Division, Fred Hutchinson Cancer Research Center, Seattle, Washington, USA; b Molecular and Cellular Biology Graduate Program, University of Washington and Fred Hutchinson Cancer Research Center, Seattle, Washington, USA; c Department of Medicine, University of Washington, Seattle, Washington, USA; d Public Health Sciences Division, Fred Hutchinson Cancer Research Center, Seattle, Washington, USA; Johns Hopkins Hospital

**Keywords:** dried blood spot, SARS-CoV-2, COVID-19, antibodies, antibody binding, neutralizing antibody, phage display, epitope mapping, neutralizing antibodies

## Abstract

Wide-scale assessment of severe acute respiratory syndrome coronavirus 2 (SARS-CoV-2)-specific antibodies is critical to understanding population seroprevalence, correlates of protection, and the longevity of vaccine-elicited responses. Most SARS-CoV-2 studies characterize antibody responses in plasma/sera. While reliable and broadly used, these samples pose several logistical restrictions, such as requiring venipuncture for collection and a cold chain for transportation and storage. Dried blood spots (DBS) overcome these barriers as they can be self-collected by fingerstick and mailed and stored at ambient temperature. Here, we evaluate the suitability of DBS for SARS-CoV-2 antibody assays by comparing several antibody responses between paired plasma and DBS from SARS-CoV-2 convalescent and vaccinated individuals. We found that DBS not only reflected plasma antibody binding by enzyme-linked immunosorbent assay (ELISA) and epitope profiles using phage display, but also yielded SARS-CoV-2 neutralization titers that highly correlated with paired plasma. Neutralization measurement was further streamlined by adapting assays to a high-throughput 384-well format. This study supports the adoption of DBS for numerous SARS-CoV-2 binding and neutralization assays.

**IMPORTANCE** Plasma and sera isolated from venous blood represent conventional sample types used for the evaluation of SARS-CoV-2 antibody responses after infection or vaccination. However, collection of these samples is invasive and requires trained personnel and equipment for immediate processing. Once collected, plasma and sera must be stored and shipped at cold temperatures. To define the risk of emerging SARS-CoV-2 variants and the longevity of immune responses to natural infection and vaccination, it will be necessary to measure various antibody features in populations around the world, including in resource-limited areas. A sampling method that is compatible with these settings and is suitable for a variety of SARS-CoV-2 antibody assays is therefore needed to continue to understand and curb the COVID-19 pandemic.

## INTRODUCTION

The ability to detect and characterize antibodies targeting severe acute respiratory syndrome coronavirus 2 (SARS-CoV-2) proteins in plasma has been one of the most informative tools during the ongoing COVID-19 pandemic. Serological testing identifies cases of previous infection, including those not revealed by symptoms and viral testing, and therefore provides a more accurate estimate of regional exposure rates than cumulative reverse transcription-PCR (RT-PCR)-based testing (1). In addition to public health surveillance, assessing SARS-CoV-2 antibody binding and functional activity has provided insight into the magnitude, features, and durability of antibody responses elicited by natural infection (2–4) and, more recently, vaccination (5–7). Antibody-based investigations have therefore informed lockdown regulations, vaccine development (8–11), possible correlates of protection (4, 12, 13), and more.

Though tremendous progress has been made to curb the pandemic, several pressing questions remain that necessitate the continued evaluation of SARS-CoV-2 antibodies in large cohorts around the world. Namely, it is unknown how long immunity conferred by infection or vaccination lasts and whether these forms of immunity are protective against viral variants. Addressing these questions will likely require global sample collection for years to come, as immunity against variants must be investigated as they emerge and there is an ever-growing list of vaccines (8–11, 14), second dose combinations (15), and boosters (16) that must be evaluated for longevity of protection.

The enormity of this task underscores the need to optimize the feasibility and practicality of SARS-CoV-2 antibody investigations. Some of the most expensive and time-consuming aspects of these studies involve the collection, shipping, and storage of plasma and serum samples. These sample types are collected via venipuncture by trained phlebotomists typically in the clinic and are immediately stored in a refrigerator or freezer and longer term at −20°C. The cold chain must be maintained during transportation for biospecimen integrity. Therefore, if individuals cannot access a clinic, phlebotomists are unavailable or overburdened, the cold chain is not maintained, or long-term −20°C storage is too costly, using plasma and/or serum sampling becomes logistically prohibitive.

Dried blood spots (DBS) have previously been used for nucleic acid and antibody testing and represent a more practical sampling type for SARS-CoV-2 antibody studies. DBS cards are prepared by spotting whole blood onto filter paper cards. Once dried, cards are stable at ambient temperature for at least a few weeks (17, 18) and can be mailed without a cold chain or special authorizations for international shipments (19). DBS cards can therefore be collected at home via a noninvasive, self-administered fingerstick and mailed to the laboratory, where small DBS discs are excised and eluted for assay use (20). DBS cards bypass the need for trained personnel for collection or the cold chain for shipping, which not only lowers costs and reduces frontline worker demand, but also facilitates sampling hard-to-reach populations.

The utility and reliability of DBS cards have been demonstrated for several decades. DBS sampling has been used for infant metabolic screening since the 1960s (21, 22) and now has broad applications, with over 2,000 analytes measured in DBS eluates to date (23). In the context of infectious disease research, DBS cards are valuable for the surveillance and study of HIV and tropical diseases (24–26) by enabling sampling in resource-limited areas. Therefore, when the current pandemic began, several groups sought to verify the use of DBS eluates for SARS-CoV-2 antibody assays. The most robust of these studies compared antibody binding measurements between paired DBS eluates and plasma or sera from COVID-19 convalescent individuals (27–36), and, impressively, all have found strong agreement between sample types. Despite this supportive evidence, the majority of these reports did not use self-collected, mailed-in fingerstick DBS cards and the compatibility of DBS with other assay formats, including neutralization, has not been carefully examined for SARS-CoV-2.

In this study, we addressed gaps in the SARS-CoV-2 DBS field by collecting paired plasma and DBS cards from COVID-19 convalescent and SARS-CoV-2-vaccinated individuals. We assessed the agreement between sample types for several antibody-based methods, including a widely used receptor-binding domain (RBD) enzyme-linked immunosorbent assay (ELISA) (37, 38), a comprehensive phage display approach (39), and a SARS-CoV-2 spike neutralization assay (40), which we optimized here for higher throughput. For all approaches, we found consistently high agreement between sample types, including between paired plasma and eluates from self-collected fingerstick DBS cards. Additionally, we evaluated the stability of antibodies on DBS cards after 6 months at room temperature (RT) and found no substantial decline in SARS-CoV-2-specific IgG binding. These results support the adoption of DBS sampling for SARS-CoV-2 antibody studies as a reliable, feasible addition to current plasma and serum approaches.

## RESULTS

### Characteristics of paired sample groups.

**FIG 1:**
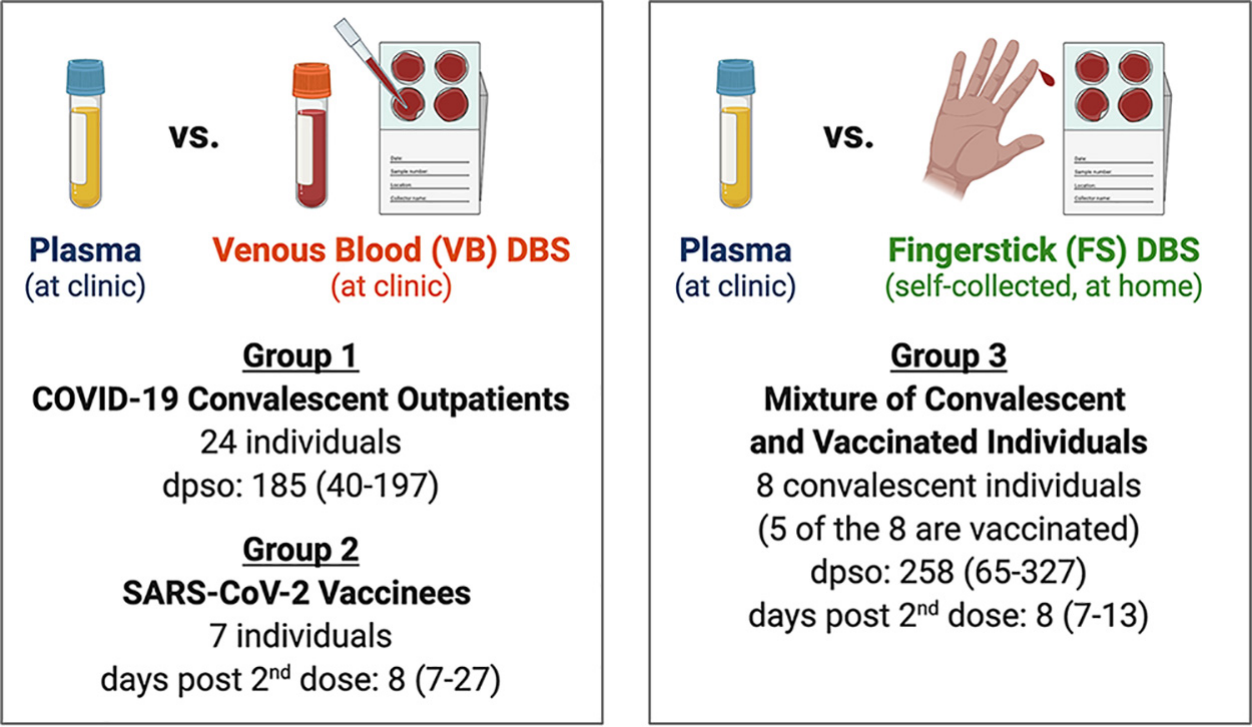
Characteristics of paired sample groups. Paired samples were assigned to one of three study groups based on the sample types collected and participant SARS-CoV-2 infection and vaccination history as outlined in the figure. All vaccinated individuals received two doses of the Moderna mRNA-1273 or Pfizer/BioNTech BNT162b2 vaccines. Values are shown as median (with range in parentheses). dpso, days post-symptom onset. The figure was created with BioRender.com.

Individuals with previous COVID-19 infection and/or SARS-CoV-2 vaccination were enrolled into a prospective observational study at the University of Washington–Seattle, and paired plasma and DBS cards were collected from these individuals. DBS cards were self-prepared at home via fingerstick (FS DBS) or at the clinic by spotting venous blood onto cards immediately after venipuncture (VB DBS). Paired samples were designated into one of three sample groups depending on DBS type and history of infection and/or vaccination, as outlined in Fig. 1.

### VB DBS eluates reflect total and SARS-CoV-2-specific IgG levels in paired plasma from COVID-19 convalescent patients.

**FIG 2:**
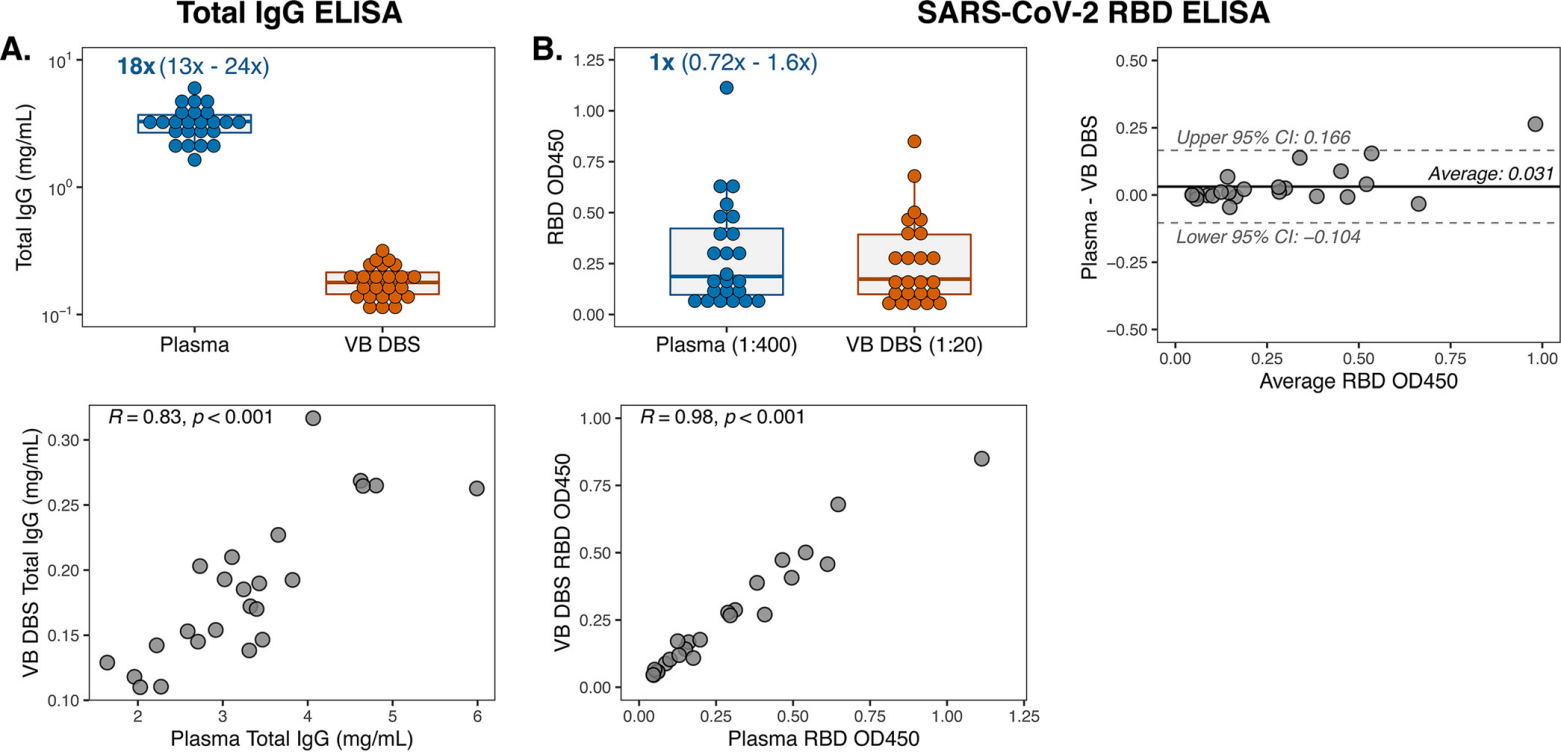
VB DBS eluates reflect total and SARS-CoV-2-specific IgG levels in paired plasma from COVID-19 convalescent patients. (A) Total IgG concentrations in group 1 VB DBS eluates and paired plasma (*n* = 24 pairs) and the Pearson *R* correlation comparing IgG levels between sample types. The median and range fold differences between paired samples are reported above the plasma data. (B) IgG reactivity to SARS-CoV-2 RBD protein at single dilutions, as indicated. Results were also depicted as a Pearson *R* correlation and a Bland-Altman plot to compare VB DBS eluate and plasma RBD OD_450_ measurements.

We first measured total IgG levels in group 1 VB DBS eluates and paired plasma (*n* = 24) to estimate the volume of plasma on each 6-mm DBS subpunch. IgG concentrations determined by ELISA ranged from 110 to 317 μg/ml in VB DBS samples eluted in 100 μl of phosphate-buffered saline-Tween (PBS-T) (Fig. 2A), which indicates that a median of 17.9 μg IgG was eluted from each disc. By comparing the IgG amount on each VB DBS disc to the levels in paired plasma, we calculated that each 6-mm disc from group 1 contains a median of 5.6 μl of plasma (range, 4.2 to 7.9 μl). Therefore, there is an approximate 20-fold initial plasma dilution introduced when DBS discs are eluted that must be accounted for in downstream assays. Overall, there was a strong correlation in IgG levels measured between these two sample types (Pearson’s *R* = 0.83) (Fig. 2A).

To determine whether VB DBS-eluted antibodies recapitulate plasma SARS-CoV-2 RBD binding, we assayed samples via a widely used in-house RBD ELISA (37, 38). At point dilutions that accounted for the initial ∼20-fold dilution introduced during DBS preparation, paired samples demonstrated very similar RBD optical density (OD) measurements (median fold change of 1) (Fig. 2B). Similarly, the trends in binding magnitude across individuals strongly agreed between sample types (Pearson *R* = 0.98) (Fig. 2B). These results demonstrate the sensitivity of DBS eluates to detect antibody binding despite the dilution that occurs due to sample processing. We further assessed eluate and plasma agreement by performing Bland-Altman analysis on the absolute difference between the OD measurements for each sample type (Fig. 2B). We observed an average bias of +0.031 OD in plasma (95% confidence interval [CI], −0.104 to 0.166) and only one sample pair, which also had the highest average OD, exceeded the 95% CIs. This average bias falls below the typical level of background observed in empty RBD ELISA wells (0.05 OD unit [data not shown]). Therefore, we found high concordance in SARS-CoV-2 RBD binding via a standard ELISA between paired VB DBS eluates and plasma from convalescent individuals.

### Polyclonal antibody response characteristics and epitope specificities can be defined using DBS.

We next sought to examine the utility of DBS to capture epitope specificity and to determine whether antibodies to the dominant SARS-CoV-2 epitopes present in polyclonal plasma are preserved in VB DBS eluates. To address this, we tested 22 sample pairs from group 1 for binding to a library of phage displaying SARS-CoV-2 peptides. This phage library expresses 39-amino-acid-long peptides that span the entire SARS-CoV-2 proteome in 20-amino-acid increments, for a total of 480 SARS-CoV-2 peptides (39). Ten micrograms of IgG for each sample was incubated with the peptide library. Antibody-phage complexes were then immunoprecipitated and sequenced to define the linear peptides that antibodies bound to in the two sample types.

**FIG 3:**
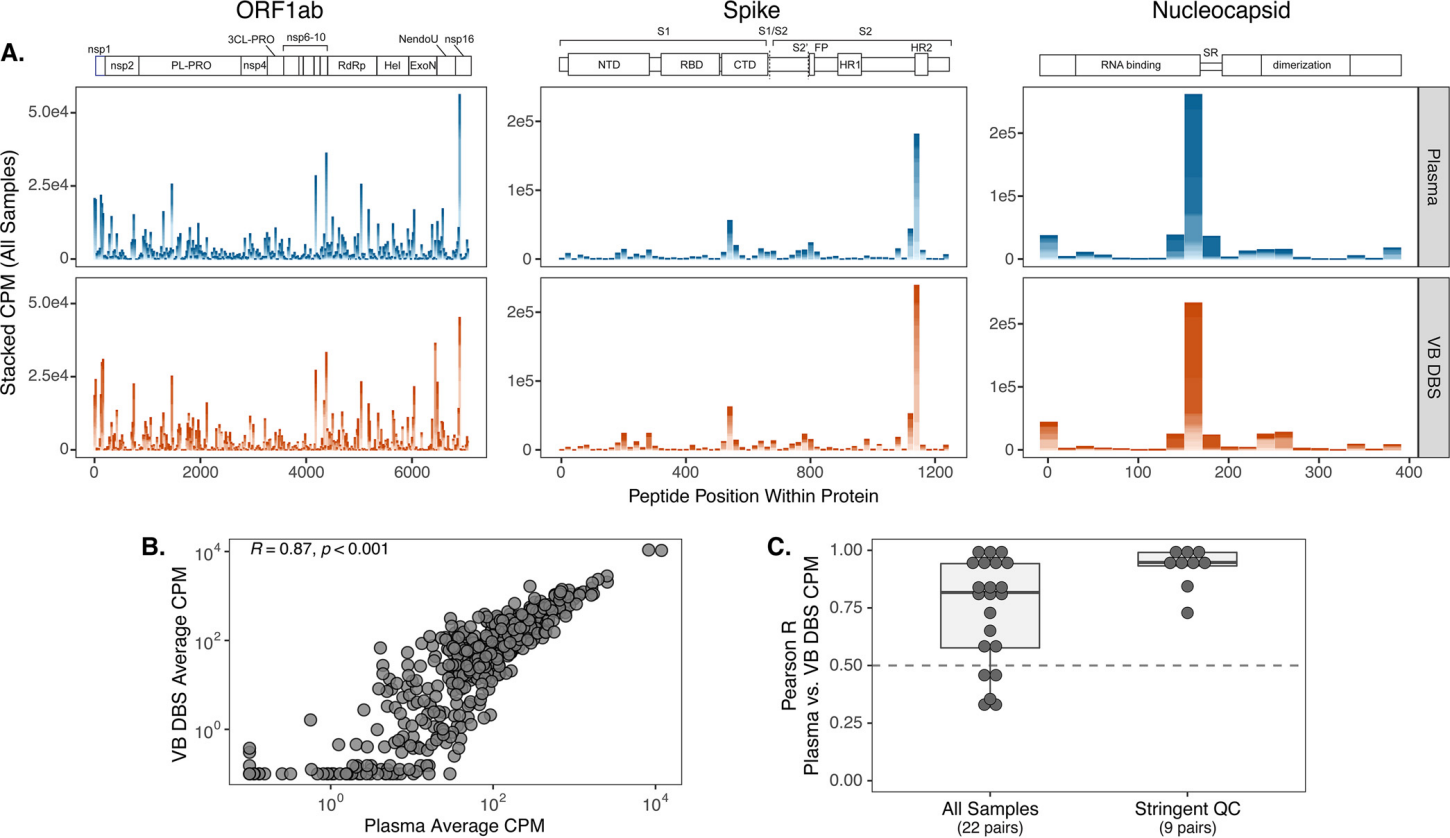
Polyclonal antibody response characteristics and epitope specificities can be defined using DBS. (A) Counts per million (CPM) of SARS-CoV-2 peptides within the three proteins with the highest magnitude of binding: ORF1ab, spike, and nucleocapsid. Peptide CPM are stacked across group 1 individuals for each sample type (*n* = 22). (B) Pearson *R* correlation of average peptide CPM between sample types. (C) CPM values between sample pairs were correlated for each individual, and the distribution of correlation coefficients is shown for all sample pairs and those with high replicate reproducibility. QC, quality control.

In agreement with a previous report from our group on convalescent plasma (39), the proteins with the highest magnitude of peptide binding as measured by counts per million (CPM) were ORF1ab, nucleocapsid, and spike for both sample types. Within these dominant sites, VB DBS eluates closely recapitulated the binding profiles observed in plasma on a cohort-wide scale (Fig. 3A). Moreover, average CPM for each SARS-CoV-2 peptide across the 22 individuals strongly correlated between sample types (Pearson *R* = 0.87) (Fig. 3B), indicating cohort-wide agreement across all proteins. To contextualize the magnitude of this correlation, we compared average CPM across the 22 plasma samples between duplicate assay wells and observed a very similar level of agreement (Pearson *R* = 0.94 [data not shown]), suggesting that antibodies eluted from DBS cards recapitulate cohort-wide plasma epitope profiling results nearly to the level of within-assay plasma replicates.

We also compared epitope profiles between VB DBS eluates and plasma on the individual level by correlating SARS-CoV-2 peptide CPM results between sample pairs. We observed strong agreement between sample pairs with a median Pearson *R* coefficient of 0.82 and 17/22 pairs having Pearson *R* values greater than 0.5 (Fig. 3C). To evaluate whether sample data quality influences agreement between paired samples, we correlated SARS-CoV-2 peptide CPM values between duplicate assay wells and focused on those sample pairs with high DBS replicate agreement (replicate Pearson *R* > 0.5; *n* = 9 pairs). Peptide counts between the resulting plasma and VB DBS pairs were consistently highly correlated (median Pearson *R* = 0.95) (Fig. 3C), which suggests that DBS eluates more closely reflect paired plasma results when DBS replicate reproducibility is high. These data support the use of VB DBS eluates in place of plasma for both cohort-wide epitope-mapping investigations and for the evaluation of individual binding profiles.

### Neutralizing antibody levels in vaccinated individuals can be accurately measured using DBS.

**FIG 4:**
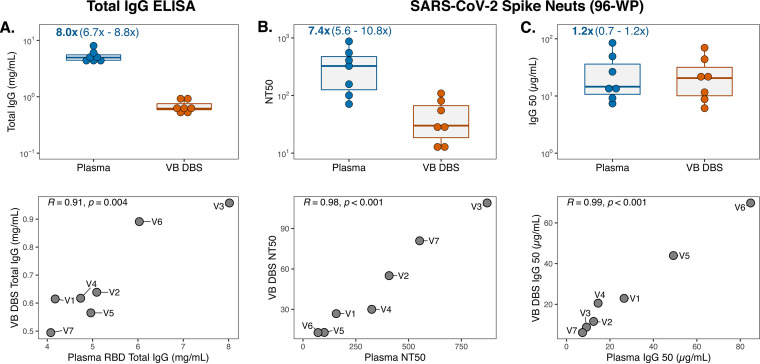
Neutralizing antibody levels in vaccinated individuals can be accurately measured using DBS. (A) Total IgG levels in concentrated, medium-eluted group 2 VB DBS eluates and paired plasma (*n* = 7 pairs) and respective Pearson *R* correlations between sample types. Data points in the correlation plot are labeled with group 2 participant IDs (V1 to V7). (B) Fifty percent neutralization titer (NT_50_) and (C) IgG_50_ (total IgG concentration divided by NT_50_) results for sample type pairs and Pearson *R* correlations. Neuts, neutralization assays.

Recent studies continue to support antibody-mediated neutralization of SARS-CoV-2 as an important immune response conferring protection against infection and COVID-19 disease (12, 13, 41). Therefore, there is a continued need to evaluate levels of neutralization in large cohorts not only to understand the duration of this response after vaccination but also to assess neutralization efficacy against emerging viral variants. To determine whether DBS eluates capture plasma neutralization activity, we collected paired plasma and VB DBS cards from seven individuals at a median of 8 days after their second Moderna mRNA-1273 or Pfizer/BioNTech BNT162b2 SARS-CoV-2 vaccination (group 2 in Fig. 1). For this experiment, DBS discs were eluted in 50 μl serum-free medium instead of 100 μl PBS-Tween to concentrate the eluate and ensure its compatibility with cell culture (see Materials and Methods). We first measured total IgG levels in these concentrated, media-eluted VB DBS eluates and paired plasma and observed a median 8-fold difference between sample types (Fig. 4A). This was approximately half of that observed with VB DBS eluates eluted in twice the volume (Fig. 2A) and corresponded with a median of 6.3 μl plasma per 6-mm disc, which was very similar to the prediction from the larger elution volume. Consistent with the total IgG results with group 1 samples, group 2 VB DBS eluates and plasma IgG levels strongly correlated (Pearson *R* = 0.91) (Fig. 4A).

To evaluate neutralization activity in plasma and DBS eluates, we leveraged a previously described SARS-CoV-2 spike-pseudotyped lentiviral particle assay (40) and found that all plasma samples from vaccinated individuals had detectable 50% neutralization titers (NT_50_; range, 71.1 to 872.1). VB DBS eluates demonstrated neutralization titers 7.4-fold lower than those of plasma (Fig. 4B), which is consistent with the 8-fold difference in total IgG levels (Fig. 4A). Remarkably, the NT_50_ values between sample types correlated very strongly (Pearson *R* = 0.98) (Fig. 4B). To understand whether the 7.4-fold difference in NT_50_ values between sample types was driven by differences in total IgG content, we calculated the IgG_50_ for each sample (IgG_50_ = IgG concentration/NT_50_). After this normalization, there was no significant difference in neutralization activity between sample types (Wilcoxon rank *P* value = 0.22, fold difference = 1.2) (Fig. 4C), and the strong correlation between sample pairs was maintained (Pearson *R* = 0.99) (Fig. 4C). Importantly, these results were not impacted by background signal from either sample type, as a prepandemic serum pool and an eluate from a blank DBS card did not yield any neutralization activity (see Fig. S1 in the supplemental material).

**FIG 5:**
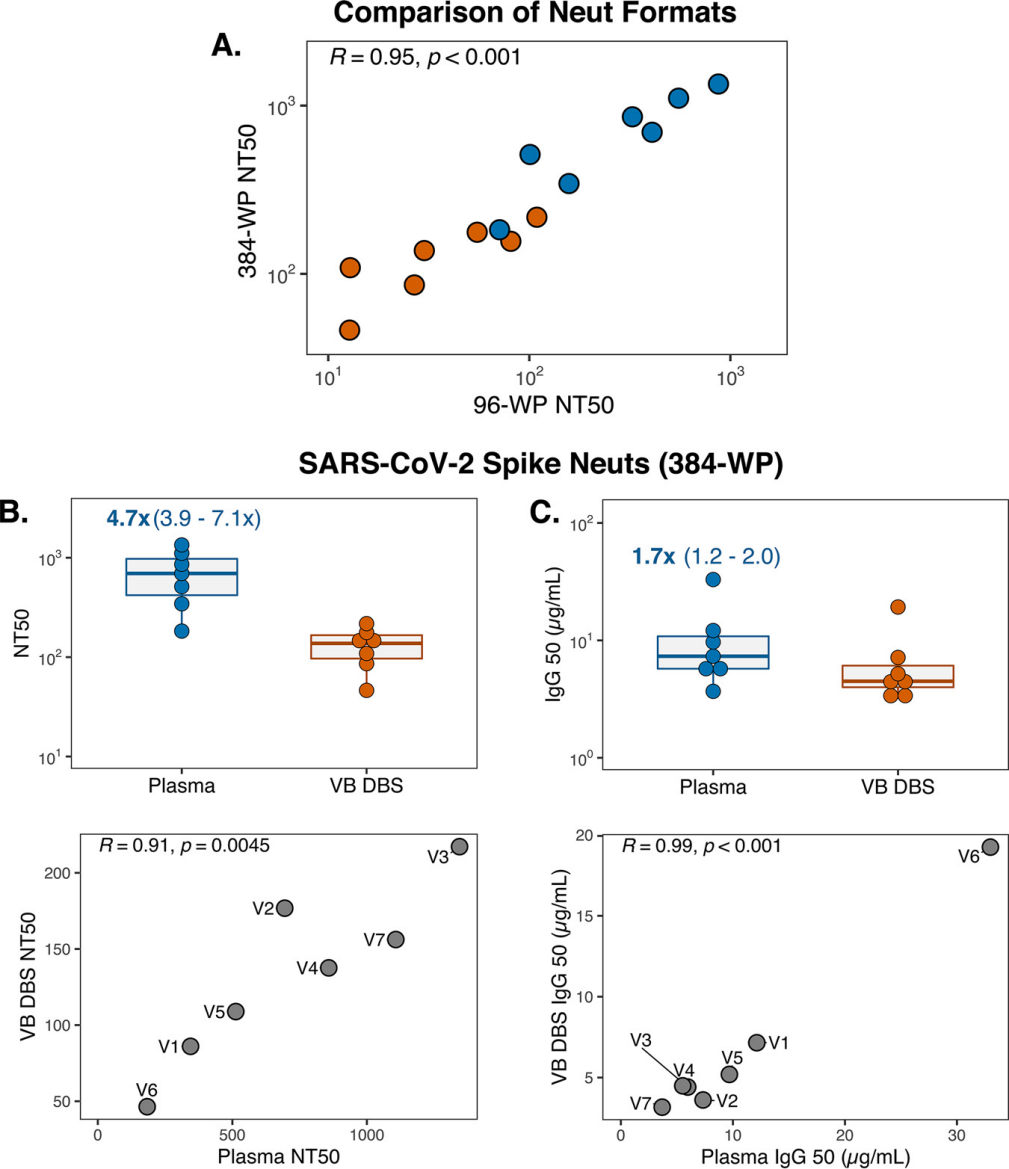
SARS-CoV-2 spike neutralization can be reliably detected in a high-throughput 384-well-plate (WP) format. (A) Pearson *R* correlation of NT_50_ measurements for group 2 plasma samples (blue) and VB DBS eluates (orange) as determined by 96-WP and 384-WP neutralization (Neut) assays. (B) NT_50_ and (C) IgG_50_ results for sample type pairs and Pearson *R* correlations in the 384-WP format. Data points in the correlation plots are labeled with group 2 participant IDs (V1 to V7). Neuts, neutralization assays.

These findings indicate that antibodies eluted from DBS cards recapitulate plasma NT_50_ trends and IgG_50_ magnitude. However, a drawback of the spike neutralization assay is that it requires large sample volumes due to its 96-well-plate (96-WP) layout, which could be limiting in the case of DBS sampling. We therefore adapted the existing SARS-CoV-2 spike neutralization assay to a high-throughput 384-WP format, which uses a third of the sample volume and assays five times the number of samples per plate. To compare assay formats, we reassayed the same seven sample pairs and found that NT_50_ measurements strongly correlated between plates for both sample types, which demonstrates that trends in neutralization titers across samples and sample types are maintained in the 384-WP format (Pearson *R* = 0.95) (Fig. 5A). Additionally, as we observed in the 96-WP assay, NT_50_ and IgG_50_ values were strongly correlated between sample types in the high-throughput format (Fig. 5B and C). The 384-WP spike neutralization assay is thus a suitable and more practical alternative to the traditional 96-WP format for both DBS eluates and plasma.

### Consistent levels of antibody recovery from DBS cards stored at room temperature for 6 months.

**FIG 6:**
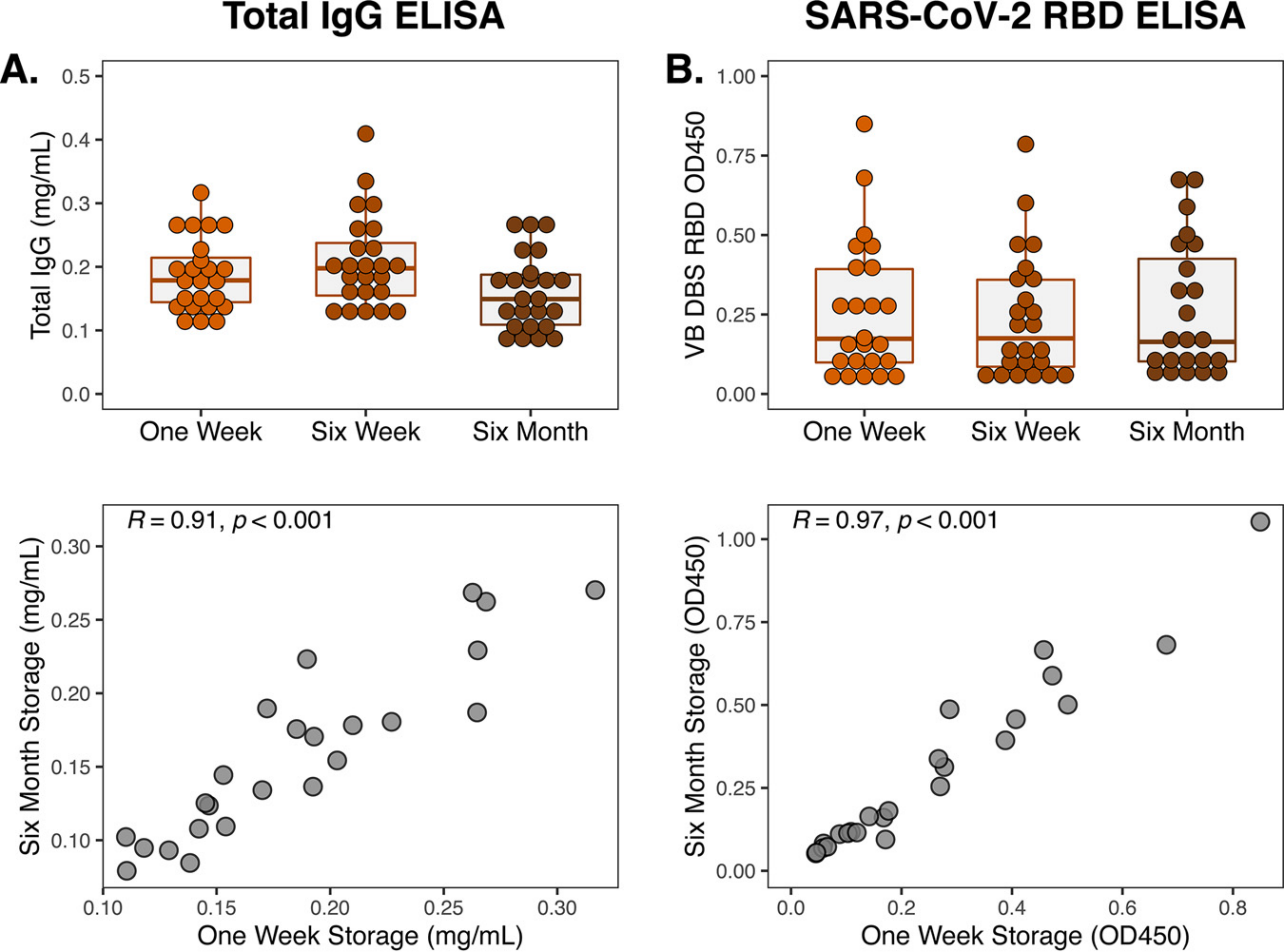
Consistent levels of antibody recovery from DBS cards stored at room temperature for 6 months. (A) Total and (B) RBD-specific IgG levels in eluates from group 1 VB DBS cards (*n* = 24) stored at room temperature for 1 week, 6 weeks, and 6 months. Pearson *R* correlation values for comparison between IgG levels measured after storage for 1 week and for 6 months are also depicted.

One of the main advantages of DBS sampling is that DBS cards do not require a cold chain for collection, transportation, or storage. To evaluate the stability of antibodies on DBS cards after prolonged storage without refrigeration or freezing, we measured total IgG and SARS-CoV-2 RBD IgG binding in eluates from group 1 VB DBS cards after 1 week, 6 weeks, and 6 months of room temperature storage (Fig. 6). DBS cards were kept in the dark in individual plastic bags with desiccant packets during this time, which is standard practice as humidity and UV light are known to damage DBS (18). Total IgG recovered from DBS was similar at 6 months to that at 1 week post-collection, and levels were highly correlated (Pearson *R* = 0.91). Likewise, SARS-CoV-2 RBD binding was consistent over this period (Pearson *R* = 0.97), demonstrating that antibodies are preserved on DBS cards stored at room temperature for at least 6 months.

### IgG binding and neutralization activity are highly correlated for self-collected FS DBS and plasma.

The DBS cards used so far in this study were prepared with venous blood, similar to prior studies (27, 30, 42–44), and were thus informative intermediates to understand antibody features that can be captured and preserved on filter paper. However, the ideal DBS sample for broad field application would be fingerstick DBS cards (FS DBS) self-collected by individuals at home and mailed to the clinic for storage, processing, and evaluation. To determine whether FS DBS eluates also generate results that reflect plasma, we collected plasma from eight individuals with prior SARS-CoV-2 infection, some of whom were also vaccinated against SARS-CoV-2 (5/8 individuals [group 3 in Fig. 1]). During their clinic visit for plasma collection, individuals were provided with a kit for FS DBS card preparation (see Fig. S2 in the supplemental material) and were instructed to fill out the card on the same day, dry it for 3 h, and mail it back to the clinic.

**FIG 7:**
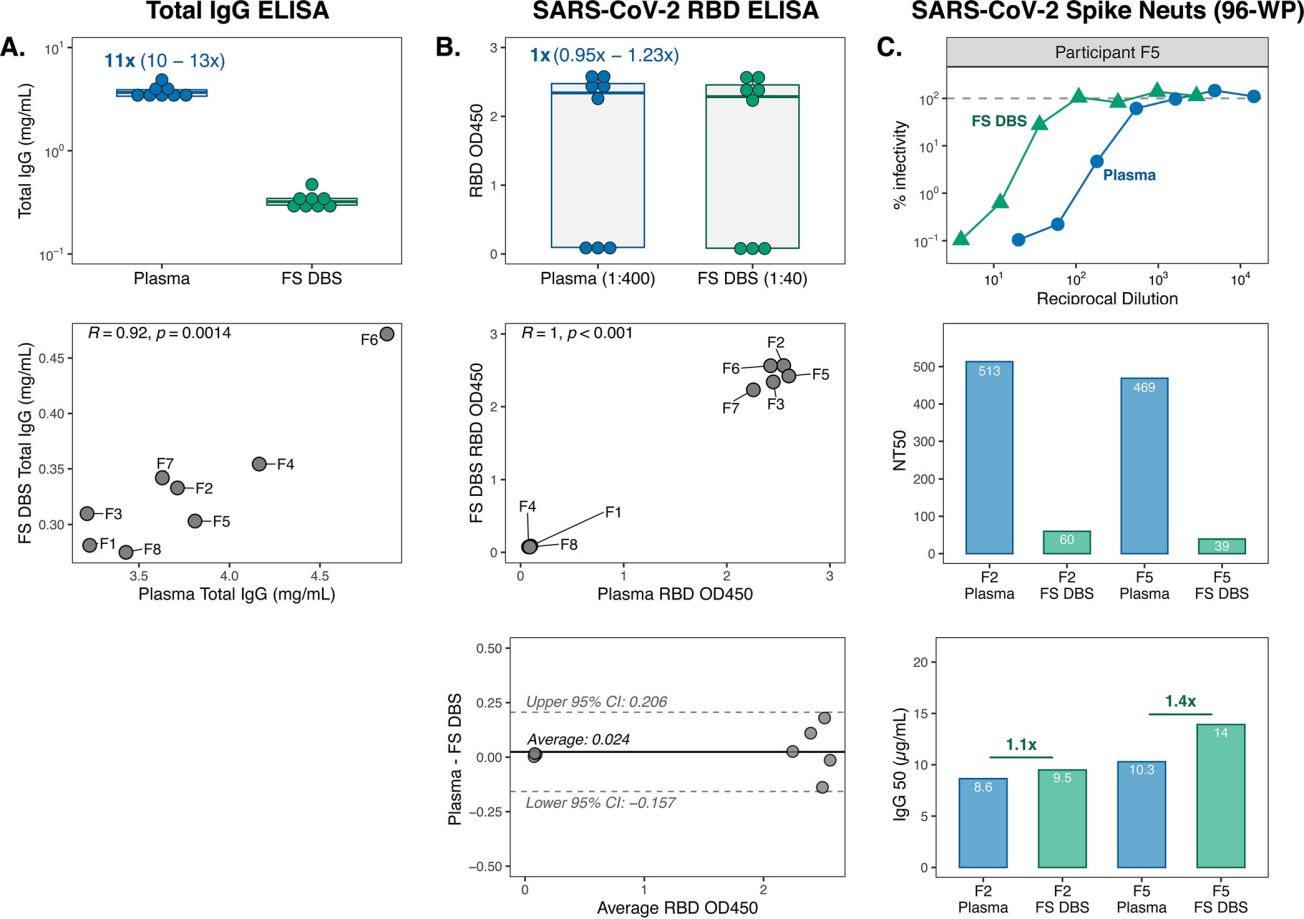
IgG binding and neutralization activity are highly correlated for self-collected FS DBS and plasma. (A) Total IgG concentrations and (B) RBD binding levels in group 3 paired sample types (*n* = 8 pairs) with respective Pearson *R* correlations and Bland-Altman plot analyses. Data points in the correlation plots are labeled with group 3 participant IDs (F1 to F8). (C) Neutralization curves against SARS-CoV-2 spike pseudovirus by plasma and FS DBS eluate from participant F5, as well as NT_50_ and IgG_50_ results for paired sample types from participants F2 and F5. Neuts, neutralization assays.

Like VB DBS samples, eluates from group 3 FS DBS cards recapitulated total IgG trends observed in paired plasma (Pearson *R* = 0.92) (Fig. 7A). We then measured RBD IgG binding in these samples, accounting for the 10-fold difference in IgG levels between sample types. As expected, there was no difference in the magnitude of RBD IgG OD measurements (Fig. 7B). These results were perfectly correlated between sample types (Pearson *R* = 1) and only demonstrated a mean bias of 0.024 OD unit. In this data set, we noticed that sample RBD binding was bimodally distributed for both sample types. This difference was driven by vaccination status, as the five individuals with detectable RBD binding all previously received SARS-CoV-2 vaccines, whereas participants F1, F4, and F8 had not.

Finally, we assessed spike neutralization activity in two group 3 participants (F2 and F5) who demonstrated high RBD binding and had enough DBS discs remaining. Though this sample size was low, the shapes of the neutralization curves were very similar between sample types (Fig. 7C), and the trend in NT_50_ values was maintained, with participant F2 having slightly higher NT_50_ than F5. IgG_50_ levels were also very similar for these two sample pairs, which suggests that FS DBS, like VB DBS, accurately reflect plasma neutralization activity.

## DISCUSSION

DBS sampling overcomes the logistical barriers of plasma collection because the cards can be self-collected by fingerstick and mailed to the laboratory at ambient temperature, where they are eluted for antibody assays. For SARS-CoV-2 studies, adoption of DBS may reduce frontline worker demand, improve sampling in resource-limited areas, and increase study enrollment. Though prior work with DBS and plasma pairs has supported the suitability of DBS for SARS-CoV-2 antibody binding assays (27–36), important gaps remained that prevented the broad implementation of this sample type. For example, most studies used DBS with venous (27, 30) or fingerstick blood collected at the clinic with the assistance of a phlebotomist (29, 31–33, 35), which does not account for variables introduced with self-sampling or ambient shipping. One study did utilize self-collected fingerstick cards (28), but only evaluated four sample pairs via an agglutination-PCR assay. Finally, all prior reports evaluated antibody binding in COVID-19 convalescent DBS samples. As vaccine distribution continues to expand and evidence grows for neutralization being an important correlate of protection (12, 13, 41), it is critical that DBS eluates also recapitulate SARS-CoV-2 neutralization activity in vaccinated individuals.

In this study, we build on previous findings that supported DBS for antibody binding by collecting and testing paired DBS and plasma from 39 individuals across a range of assays, including epitope profiling and spike neutralization assays. We evaluated not only convalescent samples but also samples from vaccinees and fingerstick DBS cards self-collected at home. The work reported here thus extends the utility of DBS sampling to ultimately support the adoption of DBS for SARS-CoV-2 antibody studies.

Prior studies often elute DBS discs in volumes that prepare samples directly for testing in a single assay format (27, 29, 31, 32, 34, 36). Because we sought to employ DBS for various assays, we first measured total IgG levels in DBS eluate and plasma pairs to estimate the volume of plasma extracted from each disc, to ultimately inform sample dilutions and data normalization approaches. Based on the total IgG results from all 39 sample pairs, we calculated that a median of 6.1 μl plasma was eluted from each 6-mm DBS subpunch. This estimate agrees with a report that compared spotted blood volume to DBS area and determined that 6-mm punches contain 5.8 to 6.4 μl plasma (27). When we accounted for the dilution factor introduced by eluting the 6.1 μl plasma from each disc into larger volumes, we found that DBS eluates recapitulated not only the trends but also the binding magnitude of paired plasma for binding and neutralization assays. These findings demonstrate that plasma and total IgG content on DBS discs can be accounted for during DBS elution, sample dilution, and/or data analysis to ensure assay sensitivity and facilitate direct comparisons between plasma and DBS results. Detection sensitivity in DBS is thus likely only compromised for assays that require undilute plasma or very large sample volumes, for which the difference in total IgG content cannot be adjusted.

Our study and prior reports demonstrate strong agreement between paired plasma and DBS for single-epitope SARS-CoV-2 binding assays, such as the commonly used RBD ELISA. However, several epitopes need to be interrogated to elucidate immunodominant proteins or the effects of variant mutations. Phage display libraries enable multiepitope investigations by providing high-throughput assessment of binding to linear peptides (45). To determine whether DBS reflect the polyclonal specificities that exist in plasma, we assayed paired samples from 22 convalescent individuals against a recently reported phage library that spans the SARS-CoV-2 proteome (39). DBS eluates identified the same immunodominant proteins as plasma and reflected plasma epitope profiles on both the cohort level and for individual participants. Individual-level agreement between plasma and DBS was further improved by only considering samples with high reproducibility between replicates, suggesting that replicate experiments may provide the best accuracy.

We also evaluated the performance of DBS in SARS-CoV-2 pseudovirus neutralization assays—the traditional 96-WP format and a new high-throughput 384-WP adaptation. In the 96-WP version, DBS replicated trends in plasma NT_50_ results and matched plasma IgG_50_ levels, demonstrating for the first time that plasma neutralization against SARS-CoV-2 spike is preserved on DBS cards. While these findings are encouraging, the 96-WP neutralization assay is not ideal for testing DBS eluates or precious plasma specimens due to large sample volume requirements. We therefore established a 384-WP format for the assessment of both plasma and DBS eluates that uses a third of the sample volume and can assay five times the number of samples per plate. The NT_50_ results strongly correlated between assay formats, and the agreement between DBS and plasma was maintained. The 384-WP SARS-CoV-2 spike neutralization assay is thus a sample-sparing, high-throughput approach for the evaluation of plasma and DBS eluates.

Our study ultimately provides evidence that SARS-CoV-2 antibody binding and neutralization are detectable in eluates from DBS cards, including those prepared at home by fingerstick and mailed to the clinic, at levels that reflect paired plasma. This work supports the implementation of DBS sampling for SARS-CoV-2 antibody investigations as a more practical alternative to plasma and serum collection, particularly in resource-limited settings.

## MATERIALS AND METHODS

### Study participants.

Paired DBS and plasma specimens were collected from COVID-19 convalescent and SARS-CoV-2-vaccinated individuals enrolled as part of the Hospitalized or Ambulatory Adults with Respiratory Viral Infections (HAARVI) research study in Seattle, WA. All participants completed informed consent as approved by the University of Washington Institutional Review Board (protocol no. STUDY00000959). Paired samples from 39 HAARVI participants were collected between September 2020 and February 2021 and were categorized into three sample groups for this study depending on the individual’s SARS-CoV-2 infection and vaccination history and the specimen types collected (Fig. 1). Group 1 consisted of 24 convalescent individuals with paired plasma and VB DBS cards. Group 2 included seven vaccinated participants with paired plasma and VB DBS cards. Finally, group 3 was comprised of eight convalescent individuals, five of whom were also vaccinated, with paired plasma and FS DBS cards. Convalescent individuals refer to those with previous PCR-confirmed, symptomatic SARS-CoV-2 infection that did not require hospitalization. Vaccinated participants received two doses of the Moderna mRNA-1273 or Pfizer/BioNTech BNT162b2 SARS-CoV-2 vaccine prior to sample collection.

### Plasma and VB DBS sample collection.

Venous blood was collected from each participant into acid citrate dextrose (ACD) tubes. For individuals in groups 1 and 2, VB DBS cards were prepared immediately after venipuncture by inverting blood tubes, pipetting 80 μl onto each circle of a Whatman 903 card (Sigma no. WHA10534612), and drying cards overnight at room temperature (RT). Dried VB DBS cards were stored in a drawer at RT in individual plastic bags containing a desiccant packet (Grainger no. 8ZF81). All plasma specimens were heat inactivated at 56°C for 1 h prior to short-term storage at 4°C or long-term storage at −80°C.

### At-home FS DBS collection kits.

Group 3 participants were provided with FS DBS collection kits and instructions at the time of an in-clinic venous blood draw and were advised to perform FS DBS sampling on the same day. Kits included the following: an instruction pamphlet (Fig. S2), one Whatman 903 card in a plastic bag with a desiccant packet, two lancets, one alcohol prep pad, one 95kPA specimen transport bag, one cardboard box, and one prepaid mailer bag. FS DBS cards were mailed to the laboratory at ambient temperature and were stored at RT under the same conditions as VB DBS cards.

### DBS elution.

Six-millimeter discs were extracted from saturated portions of VB or FS DBS cards using a biopsy punch (4MD Medical no. MLTX33-36). For FS DBS cards with variable spot sizes, discs were extracted from spots that were at least 6 mm in diameter to ensure a fully saturated sample. Forceps were used to transfer discs into a sterile 2-ml Eppendorf tube. To prevent cross-contamination, biopsy punches were replaced between cards and forceps were cleaned with 70% ethanol. PBS-T (1× PBS, 0.1% Tween 20) was added to each tube at a ratio of one disc to 100 μl PBS-T unless otherwise noted. Samples were eluted overnight at 4°C on a plate shaker with gentle agitation (70 rpm). In the morning, tubes were centrifuged at 10,000 × *g* for 5 min at RT to pellet discs and debris. Supernatants were extracted and stored at 4°C.

### Quantification of total IgG concentrations.

ELISAs were performed to determine total IgG levels in paired plasma and DBS eluates. Immulon 2HB 96-well plates (Thermo Fisher no. 3455) were coated with 50 μl of 25 μg/ml goat anti-human polyvalent IgGs (Sigma no. I1761) in 0.1 M sodium bicarbonate at 4°C overnight. Wells were washed three times with 300 μl PBS-T using a Tecan plate washer and blocked with 50 μl blocking buffer (10% [wt/vol] nonfat milk and 0.05% Tween 20 in 1× PBS) for 1 h at RT. Plasma and DBS eluates were briefly centrifuged at 10,000 × *g* for 5 min at RT to pellet debris. Sample dilutions were then prepared in blocking buffer (1:10,000 for plasma, 1:5,000 for VB DBS, and 1:10,000 for FS DBS). An IgG antibody of known concentration was diluted to 3 μg/ml and was titrated 3-fold across 10 wells to serve as the assay standard. Blocking buffer was washed from assay plates, and 50 μl of samples and standards was added to plates in duplicate. After 1 h at RT, wells were washed three times. Secondary antibody was prepared by diluting goat anti-human IgG-horseradish peroxidase (Sigma no. A0170) to 1:2,500 in blocking buffer (2.24 μg/ml). One hundred microliters was added per well, and plates were incubated at RT for 1 h. After incubation with secondary antibody and three washes, plates were developed with 50 μl of TMB (3,3′,5,5′-tetramethylbenzidine) substrate (Thermo Fisher no. 34029) and were quenched with equal volumes of 1 N sulfuric acid after 10 min. Absorbance was immediately read at 450 nm on a BioTek Epoch plate reader. Duplicate OD_450_ measurements were averaged, and IgG concentrations in plasma and DBS eluates were interpolated from the standard curve using the five-parameter logistic equation function in GraphPad Prism 8. Agreement between paired sample IgG concentrations was evaluated by calculating the Pearson correlation coefficient between sample types in RStudio.

### Detection of SARS-CoV-2 RBD IgG.

A previously described IgG ELISA against SARS-CoV-2 RBD that gained FDA emergency use authorization was adapted to measure RBD binding activity in paired plasma and DBS eluates (38). Immulon 2HB 96-well plates (Thermo Fisher no. 3455) were coated with 50 μl/well of 2 μg/ml of SARS-CoV-2 RBD protein diluted in 1× PBS. The RBD protein used as a coating was a gift from Roland Strong’s lab and was produced as previously described (46). After an overnight incubation at 4°C, plates were washed three times with 300 μl PBS-T using a Tecan plate washer and blocked with 200 μl/well of 3% (wt/vol) nonfat milk in PBS-T for 1 h at RT. Plasma and DBS eluates were centrifuged at 10,000 × *g* for 5 min at RT to pellet debris prior to being diluted in dilution buffer (1% [wt/vol] nonfat milk in PBS-T). The following point dilutions were assessed: 1:400 for participant plasma and negative-control normal human serum (Gemini Biosciences no. 100-110, lot H87WOOK), 1:20 for VB DBS eluates, and 1:40 for FS DBS eluates. RBD-specific CR3022 IgG (BEI Resources no. NR-52392) was used as a positive control at 1 μg/ml. After the blocking buffer was removed, 100 μl of sample dilutions was added in duplicate, and assay plates were incubated for 2 h at RT. Wells were washed three times with PBS-T, and 50 μl of goat anti-human IgG-horseradish peroxidase (Sigma #A0170) diluted 1:3,000 in dilution buffer (1.87 μg/ml) was added per well. After 1 h at RT, plates were washed three times with PBS-T. Fifty microliters of TMB substrate (Thermo Fisher no. 34029) per well was used to develop assay plates, and after 5 min, 50 μl of 1 N sulfuric acid was used to stop the reaction. Absorbance was immediately read at 450 nm on a BioTek Epoch plate reader. Agreement between paired sample RBD OD_450_ measurements was evaluated by calculating the Pearson correlation coefficient between sample types in RStudio.

### Linear epitope mapping via phage display, immunoprecipitation, and Illumina sequencing.

Linear CoV epitope profiling of paired plasma and VB DBS eluates was performed directly following a phage display approach previously described in detail by our group (39). Specifically, we employed the same pan-CoV phage library design and construction, IgA and IgG immunoprecipitation, Illumina library preparation, and sequence alignment techniques as the previous report. This pan-CoV phage library displays peptides 39 amino acids in length that tile across 17 CoV protein coding sequences, including the entire SARS-CoV-2-Wuhan-1 proteome (GenBank accession no. MN908947). For epitope-mapping experiments, plasma and VB DBS eluates are added to wells containing the phage library such that each well contains 10 μg of total IgG, as estimated by ELISA results. After rotating for 20 h at 4°C, phage-antibody complexes were pulled down by immunoprecipitation using a 1:1 ratio of protein A and G Dynabeads (Invitrogen no. 10002D and 10004D) and were lysed at 95°C for 10 min. Sample-selected library phage DNA was prepared for sequencing on an Illumina MiSeq with 126-bp single-end reads using the methods and primers previously described (47).

Illumina MiSeq Reporter software was used by the by the Fred Hutch Genomics Core to demultiplex peptide epitope-mapping sequencing data and generate fastq files. We then used a Nextflow data processing pipeline to align the demultiplexed sample reads to the reference peptide library in parallel, allowing for up to 2 mismatches. This pipeline builds a Bowtie index from the peptide metadata by converting the metadata to fasta format and feeding it into the bowtie-build command. The low-quality end of the reads is trimmed to 93 bp to match the reference lengths before performing end-to-end alignment and allowing for 0 mismatches. For each sample, we quantified the abundance of each peptide by using samtools-idxstats to count the number of reads mapped to each specific peptide in the reference library. Counts per million (CPM) values were calculated from raw read counts to control for read depth differences between samples. Peptide CPMs were merged into a count matrix organized by unique identifiers for each peptide and sample. The metadata tables were tied with the count matrix into an xarray data set using shared coordinate dimensions of the unique sample and peptide identifiers. We used this data set organization as the starting point for downstream epitope profiling analyses.

Due to the focused nature of this investigation on whether DBS agrees with plasma for SARS-CoV-2 antibody responses, downstream analyses specifically evaluated the 480 SARS-CoV-2 peptides included in the library. The following analyses were performed in R to compare plasma and VB DBS linear epitope profiling results. (i) Pearson correlation coefficients were determined for CPM between within-assay technical replicates to inform data quality. (ii) CPM for within-assay replicates were then averaged, and participant-level agreement between sample types was assessed by comparing CPM for VB DBS and plasma pairs via Pearson correlation. (iii) Cohort-level agreement between sample types was evaluated by calculating the average CPM for each peptide across all plasma or VB DBS samples and determining the Pearson correlation coefficient between sample type averages.

### SARS-CoV-2 spike pseudovirus production.

Pseudovirus expressing SARS-CoV-2 spike protein was produced, and the titer was determined as previously described (40). HEK293T cells were added to 6-well plates at 5 × 10^5^ cells per well in Dulbecco’s modified Eagle’s medium (DMEM) supplemented with 10% fetal bovine serum, 2 mM l-glutamine, and penicillin/streptomycin/amphotericin B (Fungizone). After 16 to 25 h, cells were transfected using FuGENE-6 (Promega no. E2692) with the Luciferase_IRES_ZsGreen backbone, Gag/Pol, Rev, and Tat lentiviral helper plasmids, and a plasmid containing the codon-optimized spike sequence from the Wuhan-Hu-1 strain, which contained a 21-amino-acid deletion at the cytoplasmic tail (also known as HDM-SARS2-Spike-delta21). After 24 h, medium was replaced with fresh supplemented DMEM. Between 50 and 60 h posttransfection, viral supernatants were collected, filtered through 0.22-μm-pore Steriflip filters, concentrated using 100-kDa Amicon filters (EMD Millipore no. UFC910024), and stored at −80°C. The titer of pseudovirus was determined by seeding 96-well black-walled plates with 1.25 × 10^4^ HEK293T-ACE2 cells and, after 16 to 24 h, adding 100 μl of undiluted viral supernatant per well in duplicate. Viral supernatants were then diluted 2-fold in supplemented DMEM over eight wells. Vesicular stomatitis virus glycoprotein (VSV-G) and no-viral-entry-protein (VEP) positive and negative controls, respectively, were included on each plate and treated the same as spike pseudovirus, except that VSV-G titration started at a 1:10 dilution. After 60 h, 100 μl per well was removed, and 30 μl of Bright-Glo (Promega no. E2620) was added. Relative luciferase units (RLU) were measured on a LUMIstar Omega plate reader (BMG Labtech).

### SARS-CoV-2 spike neutralization assays (96-WP and 384-WP).

DBS samples were eluted for neutralization assays with 50 μl of serum-free supplemented DMEM to ensure their compatibility with cell culture. SARS-CoV-2 spike neutralization assays in the traditional 96-WP format were carried out according to a previous report (40). HEK293T-ACE2 cells were brought to 2.5 × 10^5^ cells/ml, and 96-well black-walled plates were seeded with 50 μl. Assay plates also contained four wells seeded with HEK293T cells without ACE2 and four wells without any cells as negative controls. After 12 to 16 h, 60 μl of plasma and DBS dilutions were prepared in a separate round-bottomed 96-WP plate with serum-free supplemented DMEM, due to the fact that DBS were eluted in this medium. Samples were initially diluted 1:20 and 1:4 for plasma and DBS, respectively, and were then diluted 3-fold over seven wells. Duplicate dilution wells were prepared for each sample titration. Spike pseudovirus was then diluted to (3.3 to 5) × 10^5^ RLU per ml in supplemented DMEM, and 60 μl was mixed with the prepared plasma and DBS titrations. After 1 h at 37°C, 100 μl from the virus/sample wells was transferred to the cell plate. To read the plates, 100 μl of medium was removed from each well approximately 52 to 58 h postinfection, and 30 μl of Bright-Glo was added. After 2 min, RLU was measured on a LUMIstar Omega plate reader. Technical replicate RLUs were averaged, and the percentage of infectivity was calculated by dividing sample RLU by the corresponding row’s positive-control RLU value, which was from virus plus cells from wells that did not have sample added. The 50% neutralization titers (NT_50_) were determined using GraphPad Prism 8’s inhibitor versus response curve, with top and bottom parameters constrained to 1 and 0, respectively. Finally, IgG_50_ values were calculated by dividing each sample’s total IgG concentration by its NT_50_. Agreement between paired sample measurements for NT_50_ and IgG_50_ values was evaluated by calculating the Pearson correlation coefficient between sample types in RStudio.

Spike neutralization assays were adapted to 384-WPs using the same incubation times, sample dilutions, and reagents as described for the 96-WP assay, but with working volumes reduced to approximately 30%. Briefly, black-walled 384-WPs were seeded with 15 μl of HEK293T-ACE2 cells at a concentration of 2.5 × 10^5^ cells/ml. For negative controls, eight wells were seeded with HEK293T cells that do not express ACE2, and an additional eight wells contained supplemented DMEM only. After 12 to 16 h, sample dilutions were prepared in round-bottomed 96-WPs with a total volume of 36 μl per well. Spike pseudovirus was diluted to (3.3 to 5) × 10^5^ RLU per ml, and 36 μl was mixed into sample dilution wells. After 1 h at 37°C, 30 μl from each virus/sample well was transferred to two wells on the 384-well assay plate. Roughly 52 to 58 h postinfection, plates were read by removing 30 μl of medium per well, adding 9 μl of BrightGlo per well, and measuring RLU on a LUMIstar Omega plate reader after 2 min.

### Data availability.

The Nextflow pipeline, used to align epitope-mapping sample reads to the reference library, is available at https://github.com/matsengrp/phip-flow. The custom RStudio code generated and used in this study to perform statistical analyses and visualize data is available upon request. Any additional information required to reanalyze the data reported in this paper is also available upon request.
