# Supplementary material for: SARS-CoV-2 Antibody Binding and Neutralization in Dried Blood Spot Eluates and Paired Plasma
Source: Microbiol Spectr. 2021 Oct 20;9(2):e01298-21. doi: 10.1128/Spectrum.01298-21 (PMC8528110; doi:10.1128/Spectrum.01298-21)
Supplement: SUPPLEMENTAL FILE 1 — Supplemental material. Download SPECTRUM01298-21_Supp_1_seq1.pdf, PDF file, 1.0 MB [file spectrum01298-21_supp_1_seq1.pdf]

SUPPLEMENTAL MATERIAL

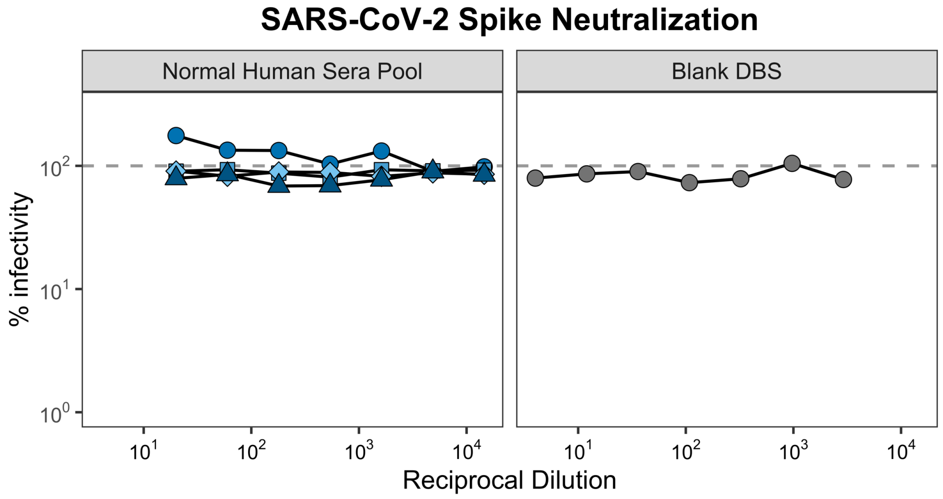

Figure S1. Undetectable levels of spike neutralization in negative control DBS and plasma. A pre-pandemic normal human sera pool (n=4 assay days) and the eluate from a blank DBS card were tested for SARS-CoV-2 spike neutralization activity at the same dilutions as participant samples, according to sample type.

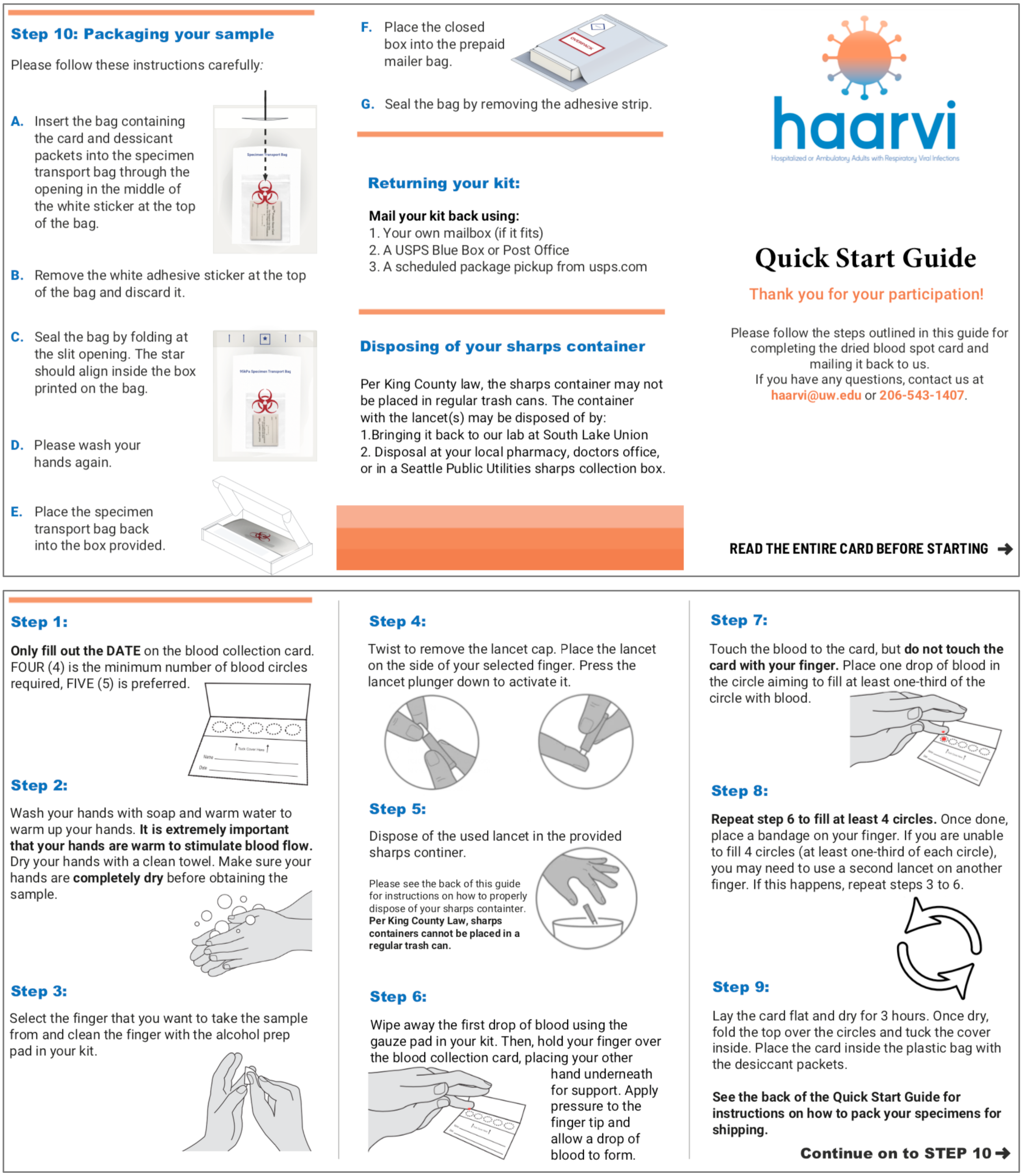

Step 10: Packaging your sample

Please follow these instructions carefully:

A. Insert the bag containing the card and dessicant packets into the specimen transport bag through the opening in the middle of the white sticker at the top of the bag.

B. Remove the white adhesive sticker at the top of the bag and discard it.

C. Seal the bag by folding at the slit opening. The star should align inside the box printed on the bag.

D. Please wash your hands again.

E. Place the specimen transport bag back into the box provided.

F. Place the closed box into the prepaid mailer bag.

G. Seal the bag by removing the adhesive strip.

Returning your kit:

Mail your kit back using:
1. Your own mailbox (if it fits)
2. A USPS Blue Box or Post Office
3. A scheduled package pickup from usps.com

Disposing of your sharps container

Per King County law, the sharps container may not be placed in regular trash cans. The container with the lancet(s) may be disposed of by:
1.Bringing it back to our lab at South Lake Union
2. Disposal at your local pharmacy, doctors office, or in a Seattle Public Utilities sharps collection box.

haarvi

Hospitalized or Ambulatory Adults with Respiratory Viral Infections

Quick Start Guide

Thank you for your participation!

Please follow the steps outlined in this guide for completing the dried blood spot card and mailing it back to us.
If you have any questions, contact us at haarvi@uw.edu or 206-543-1407.

READ THE ENTIRE CARD BEFORE STARTING →

Step 1:

Only fill out the DATE on the blood collection card. FOUR (4) is the minimum number of blood circles required, FIVE (5) is preferred.

Step 2:

Wash your hands with soap and warm water to warm up your hands. **It is extremely important that your hands are warm to stimulate blood flow.** Dry your hands with a clean towel. Make sure your hands are **completely dry** before obtaining the sample.

Step 3:

Select the finger that you want to take the sample from and clean the finger with the alcohol prep pad in your kit.

Step 4:

Twist to remove the lancet cap. Place the lancet on the side of your selected finger. Press the lancet plunger down to activate it.

Step 5:

Dispose of the used lancet in the provided sharps continer.

Please see the back of this guide for instructions on how to properly dispose of your sharps containter. **Per King County Law, sharps containers cannot be placed in a regular trash can.**

Step 6:

Wipe away the first drop of blood using the gauze pad in your kit. Then, hold your finger over the blood collection card, placing your other hand underneath for support. Apply pressure to the finger tip and allow a drop of blood to form.

Step 7:

Touch the blood to the card, but **do not touch the card with your finger.** Place one drop of blood in the circle aiming to fill at least one-third of the circle with blood.

Step 8:

Repeat step 6 to fill at least 4 circles. Once done, place a bandage on your finger. If you are unable to fill 4 circles (at least one-third of each circle), you may need to use a second lancet on another finger. If this happens, repeat steps 3 to 6.

Step 9:

Lay the card flat and dry for 3 hours. Once dry, fold the top over the circles and tuck the cover inside. Place the card inside the plastic bag with the desiccant packets.

See the back of the Quick Start Guide for instructions on how to pack your specimens for shipping.

Continue on to STEP 10 →

Figure S2. Instruction pamphlet for self-collection of fingerstick DBS cards. Study participants were provided with the depicted instructions and materials during a clinic visit where a blood sample was collected.
